# Treatment of refractory epilepsy with natalizumab in a patient with multiple sclerosis. Case report

**DOI:** 10.1186/1471-2377-10-84

**Published:** 2010-09-23

**Authors:** Stefano Sotgiu, Maria R Murrighile, Gabriela Constantin

**Affiliations:** 1 Department of Neuroscience, Section of Neurology, University of Sassari, Viale San Pietro 10, 07100 Sassari, Italy; 2 Department of Pathology and Diagnostics, Section of General Pathology, University of Verona, Strada le Grazie 8, 37134 Verona, Italy

## Abstract

**Background:**

Multiple sclerosis (MS) is considered an autoimmune disease of the central nervous system and therapeutic inhibition of leukocyte migration with natalizumab, an anti-alpha4 integrin antibody, is highly effective in patients with MS. Recent studies performed in experimental animal models with relevance to human disease suggested a key role for blood-brain barrier damage and leukocyte trafficking mechanisms also in the pathogenesis of epilepsy. In addition, vascular alterations and increased leukocyte accumulation into the brain were recently documented in patients with refractory epilepsy independently on the disease etiology.

**Case report:**

Here we describe the clinical course of a 24-year-old patient with MS in whom abrupt tonic-clonic generalized seizures manifested at disease onset. Although MS had a more favorable course, treatment with glatiramer acetate and antiepileptic drugs for 7 years had no control on seizure generation and the patient developed severe refractory epilepsy. Interestingly, generalized seizures preceded new MS relapses suggesting that seizure activity may contribute to MS worsening creating a positive feedback loop between the two disease conditions. Notably, treatment with natalizumab for 12 months improved MS condition and led to a dramatic reduction of seizures.

**Conclusion:**

Our case report suggests that inhibition of leukocyte adhesion may represent a new potential therapeutic approach in epilepsy and complement the traditional therapy with anti-epileptic drugs.

## Background

Multiple sclerosis (MS) is considered a T cell-mediated autoimmune disease of the central nervous system (CNS) with a complex genetic background [1]. It is accepted that blood-brain barrier (BBB) breakdown and T cells migration across BBB initiate an immune response against CNS myelin antigens and contribute to disease pathogenesis [2,3]. In addition, degeneration including loss of axons, diffuse damage to normal appearing white matter and involvement of deep and cortical gray matter contribute substantially to the disability progression [1]. Clinically, the focal myelin and neuronal destruction leads to a variety of relapsing-remitting symptoms, which later in the course may become persistent or progressive [4].

Seizures can occur in MS patients and the risk of epilepsy seems to be three-times higher in patients with MS than in the general population [5]. Seizures can be the presenting symptom of MS but have been observed in relapsing-remitting as well as in secondary or primary progressive MS. β-interferons, which are often used for the treatment of MS, may have pro-convulsant effects [6]. Moreover, MS symptoms can be aggravated by several antiepileptic drugs (AEDs), which can mimic disease activity [5]. Up to now, no clinical trials for the treatment of epilepsy in MS patients have been performed and, therefore, no clear recommendations can be given.

Recent evidence suggests that inflammation mechanisms play a role in the pathogenesis of epilepsy [7-12]. Moreover, recent studies performed in an experimental mouse model of epilepsy suggested that leukocyte trafficking mechanisms induce BBB damage leading to seizure generation [10]. These results were supported by studies performed in an acute viral meningitis model in which cytotoxic T lymphocytes and massive recruitment of monocytes and neutrophils were required for vascular leakage and seizure-induced death [11]. Importantly, white matter angiopathy and increased number of CD68-positive cells and CD3-positive T cells in perivascular cavities were documented in a subpopulation of young patients with refractory epilepsy [12]. In addition, increased number of leukocytes was observed in brain parenchyma of epileptic patients, independently on the disease etiology [10]. However, despite growing evidence showing a role for leukocyte trafficking and BBB damage in seizure generation, clinical trials with anti-adhesion therapies have not been performed yet in patients with epilepsy.

Current anti-inflammatory and immunosuppressive MS-treatments include β-interferons, glatiramer acetate (GA) and different chemotherapies. Recently, natalizumab, a monoclonal antibody directed against the α4 chain of integrin VLA-4, an adhesion molecule controlling leukocyte adhesion to brain endothelium, was approved by the U.S. Food and Drug Administration and the European Medicines Agency as monotherapy for highly active relapsing-remitting MS. Despite the occurrence of progressive multifocal leukoencephalopathy (PML) as adverse reaction, natalizumab represents the most potent drug approved thus far for the treatment of relapsing-remitting MS [13,14].

Here we describe a dramatic reduction of seizures after treatment with natalizumab in a patient with severe refractory epilepsy and MS. For better clarity we split the description of seizures and epilepsy from the non-epileptic MS course.

## Case presentation

The 10-years follow-up, from MS onset (October 1999) to January 2010 is summarised on Table 1. Clinical (relapse and EDSS) and subclinical (MRI) MS activities are indicated. Epilepsy-related information concerning type, severity (*status epilepticus*) and frequency of seizures, type and dose of antiepileptic drugs (AEDs) are also reported. In the time interval 1999-2000 the patient suffered from 1 partial and 1 generalised seizure (those at disease onset) as described below in the text. Abbreviations and symbols are the followings: n.d. = not done; * = mild decrease in mentation; ** = marked decrease in learning and memory; EDSS = Kurtzke's expanded disability status scale; VPA = valproate; CBZ = carbamazepine; LMT = lamotrigine; TOP = topiramate; OXC = oxcarbazepine; KEP = levetiracetam; GBP = gabapentin; ^§ ^= LMT discontinued after 1 month for allergic dermatitis. Each non-epileptic MS relapse has been treated with methyl-prednisolone 1 gr/daily for 3-5 days without tapering-off.

**Table 1:** Clinical follow-up of the MS case from MS onset (1999) up to January 2010. Abbreviations and symbols in the text (Case Presentation)

Year	1999-2000	2001	2002	2003	2004	2005	2006	2007	2008	2009-2010
**Non-epileptic MS relapses**	1	0	1	0	1	0	0	1	1	0
**Brain MRI activity** *(new T2 or Gd^+ ^lesions or both)*	+	-	+	-	-	n.d.	-	n.d.	+	-
**EDSS score**	-	0	1	1	2	2	2.5*	3*	4**	2.5*
**MS therapy**	-	-	GA	GA	GA	GA	GA	GA	GA	Natalizumab
**Partial seizures**	1 (onset)	1	4	4	6	8	7	8	10	4
**Generalized seizures**	1 (onset)	1	3	2	3	6	5	5	7	0
***Status epilepticus***	0	0	0	0	0	1	0	1	1	0
**AEDs **(daily dose in mg*)*		VPA 600	VPA 1000	VPA 1000	VPA 1000	VPA 1300	VPA 1000	KEP 3000	KEP 3000	KEP 1000
			CBZ 300	CBZ 800	CBZ 1200	CBZ 1400	LMT^§^	OXC 2100	OXC 2400	GBP 900
							TOP 250		GBP 900	

### MS onset and course

#### a) From onset (1999) to GA therapy (2002)

In October 1999, a previously healthy 24-year-old man complained of the sudden appearance of a brainstem syndrome (multiple oculomotor nerves involvement and gait instability) followed, 24 hours later, by an abrupt tonic-clonic generalized seizure. Routine laboratory exams were normal. Family and personal history were all negative for neuro-psychiatric, hereditary and autoimmune diseases. MRI showed multiple supra-and infra-tentorial T2-w hyper-intense and two Gd-enhanced T1-w lesions. Cerebrospinal fluid (CSF) composition was normal in protein level and cell count. Post-critical EEG was also normal.

Differential diagnosis was made with conditions causing seizures and mimicking MS either of inflammatory (vasculitis, sarcoidosis, systemic lupus erythematosus, Sjögren's syndrome, Behçet's disease), infectious (herpes, Lyme disease, HTLV-1, syphilis), genetic (CADASIL, adrenoleukodystrophy, lysosomal and mitochondrial disorders), metabolic (vitamin B12 deficiency, hyper-homocysteinemia), and neoplastic origin (CNS lymphoma). Anti-voltage-gated potassium channel and onconeural antibodies for limbic encephalitis were also tested. All investigations performed to seek for signs of these diseases resulted negative. A subsequent spinal MRI performed a week after disease onset showed cervical T2-w hyper-intense lesions with no Gd-enhancement. Six CSF oligoclonal bands were found, which allowed a diagnosis of laboratory-supported definite MS according to the Poser criteria in use at that time [4]. A course of methyl-prednisolone 1 gr/day for five days was administered, followed by a long-term remission of all symptoms and signs. During 2000 and 2001 the patient did not complain of any symptom referable to MS, except from two seizures (see below). During 2000/2001 Kurtzke's EDSS (expanded disability status scale) score [15] was zero and brain MRI in 2001 was unchanged. The patient received no treatment during this period.

#### b) MS course during GA treatment (2002-2008)

The first MS relapse occurred during 2002 and was characterized by a mild cerebellar ataxia and dysmetria (Table 1). Romberg sign was absent. A new brain MRI showed the occurrence of new small T2-w lesions. A short course of methyl-prednisolone 1 gr/day for three days was administered. As β-interferons seem contraindicated for their detrimental effect on seizures [6], treatment with GA was started and was well tolerated till the end of 2008. After six months of GA treatment the EDSS score was 1 (very mild dysmetria on left hand).

The second MS relapse occurred in May 2004 and was characterized by diplopia. Neurological examination disclosed a mild right internuclear ophthalmoparesis and MRI revealed the absence of new T2-w or Gd-enhancing lesions (Table 1). A new course of methyl-prednisolone 1 gr/day for five days was administered and symptoms subsided in two week. After 6 months (November 2004) the EDSS score was 2 (dysconjugated nystagmus with diplopia and dysmetria).

The third non-epileptic MS relapse occurred three years later (April 2007) and was characterized by urinary urgency. MRI was not performed. At examination, a left Babinski sign was evident together with nystagmus and dysmetria. EDSS score was 3 (Table 1). A new course of methyl-prednisolone 1 gr/day for five days was administered and symptoms subsided in four weeks.

**Figure 1:**
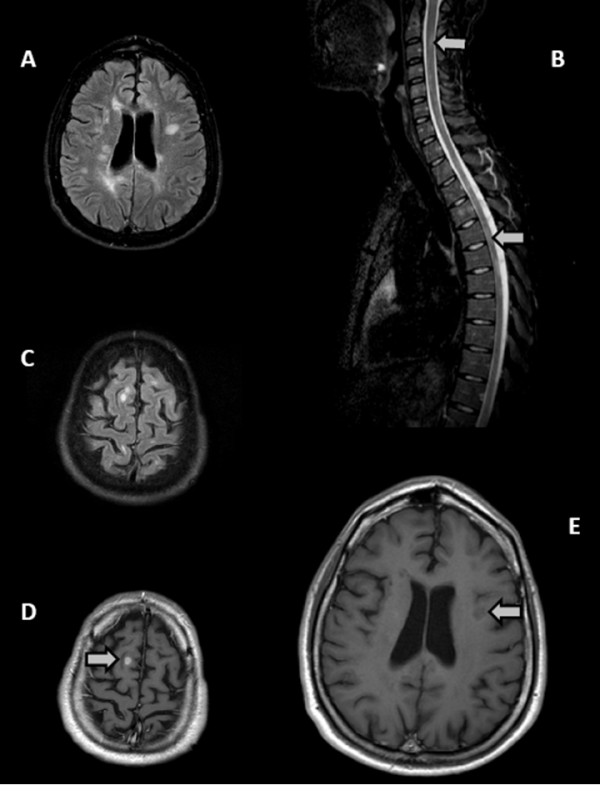
**Cranial and spinal MRI before and after treatment with Natalizumab**. **A**. Axial FLAIR (fluid attenuation inversion recovery) scan performed on October 2008 before Natalizumab treatment showing multiple hyperintense MS lesions in the periventricular areas of both hemisheres. **B**. Sagittal STIR (Short TI Inversion Recovery) sequence of spinal cord performed on October 2008, showing a high number of hyperintense demyelinating lesions in the cervical and dorsal spinal tracts (two representative are indicated by arrows). **C**. Axial FLAIR scan performed on October 2008 showing hyperintense subcortical MS lesions of both hemisheres. **D**. After intravenous injection of Gadolinium, an intense contrast enhancement is shown in one subcortical lesion of the right parietal area (arrow). **E**. Representative axial T1-wheighted scan (periventricular section) after intravenous injection of Gadolinium, performed on October 2009 during natalizumab treatment. Multiple hypointense MS lesions are evident in the periventricular areas of both hemispheres (the largest is indicated by the arrow) with no Gadolinium-enhancement. The MRI evaluation also included T2 and FLAIR scans of the whole brain. Besides the absence of contrast-enhancement, no new or enlarging T2-FLAIR lesions were evident after natalizumab treatment (not shown).

In October 2008 new clinical (right hemiparestesia) and subclinical MRI activities emerged, showing new T2-w lesions in both brain and spinal cord and Gd-enhancing T1-w brain lesions (Figure 1A-D). Patient's reflexes were enhanced in both legs and pallesthesia was absent in the feet. In addition memory, attention, language, perception and problem solving were markedly reduced, and EDSS score increased to 4. At this point, the clinical and MRI findings indicated the need for a new MS-therapy and natalizumab treatment was planned to begin in 2009 after three months wash-out of GA.

#### c) Effect of Natalizumab on MS course

Natalizumab was administered at a dose of 300 mg every 4 weeks from February 2009 till January 2010 (12 months follow-up, present study). The cognitive status improved and the EDSS score decreased to 2.5 in January 2010 (Table 1). He did not complain side effects of natalizumab and the JCV detection in blood after 9 months of treatment was negative. The MRI in October 2009 showed neither Gd-enhancement nor new or enlarging T2-w lesions (Figure 1E).

### 2. Epilepsy onset and course

#### a) Epilepsy onset

**Figure 2:**
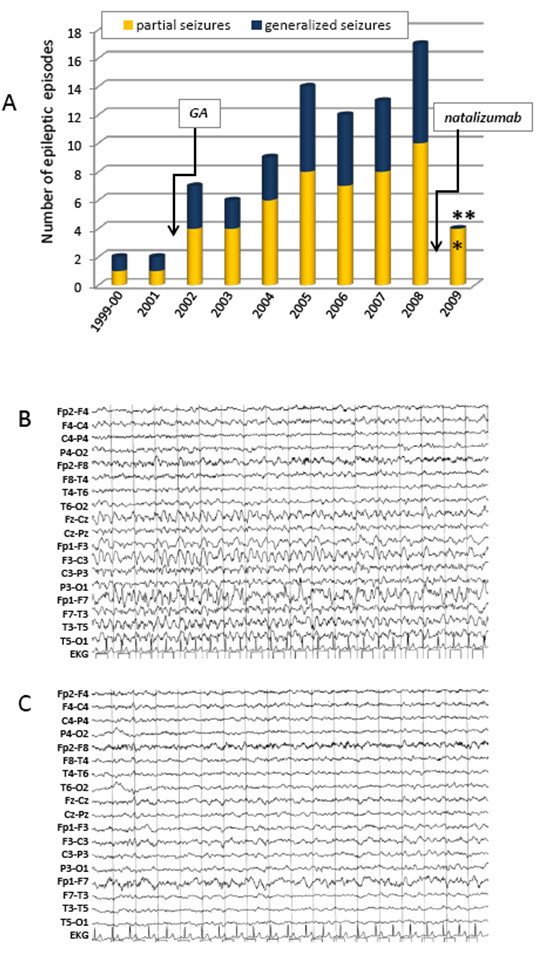
**Effect of Natalizumab on seizure frequency and EEG tracks**. **A**. Bars indicate the number of episodes of partial (yellow) and generalized (blue) seizures along the 10-years period. After natalizumab introduction (arrow), the number (Y axis) of partial seizure dramatically dropped from 10 to 4 (60% reduction), while generalized seizures disappeared. T-test (two-tailed 95% confidence interval) was used to test the null hypothesis (number of seizures after the start of natalizumab is not different from the mean of the 2002-2008 period). Mean values were 6.71 ± 2.21 (95% CI 3.22-5.62) for partial seizures and 4.43 ± 1.81 (95% CI 5.24-8.17) for generalized seizures, respectively. One tailed p-values are 0.003 for partial (*) and 0.00004 for generalized (**) seizures, allowing to strongly reject the null hypothesis. **B**. Intercritical scalp EEG recording (18'' duration) of the case, performed while awake on July 2008 and showing a reactivation of a focus of sharp waves and low-amplitude slow activity over the left fronto-temporal region, which correspond to a complex partial seizure accompanied by gestural automatisms. The patient was on therapy with GA and on poly-AEDs treatment (oxcarbazepine 2400 mg/day, levetiracetam 3000 mg/day and gabapentin 900 mg/day). **C**. Intercritical scalp EEG recording (18'' duration) performed on June 2009 while awake, showing minimal low-amplitude slow activity over the left fronto-temporal region. The patient was on therapy with natalizumab and on poly-AEDs treatment with levetiracetam 2000 mg/day (33% reduction) and gabapentin 900 mg/day. Scalp EEG recording set up: PA filter 0.53 Hz; PB filter 30 Hz, amplitude 70 microvolt/cm. Electrode placement refers to the Jasper's 10-20 system. EKG = electrocardiogram.

In October 1999, 24 hours after the sudden appearance of the demyelinating brainstem syndrome the patient presented a generalized tonic-clonic epileptic seizure with tongue laceration and urinary incontinence. Post-critical EEG (24 hours after seizure) was normal. No specific treatment for seizures was initiated. Although EDSS score was zero and brain MRI was unchanged, on December 2001 the patient presented a second generalized tonic-clonic epileptic seizure, which was preceded by a simple partial seizure of focal motor activity on the left body part (Table 1, Figure 2A). Post-ictal EEG showed a focus of low-amplitude slow activity over the left fronto-temporal region. Antiepileptic therapy with Valproate (VPA, 600 mg/day) was started.

#### b) Epilepsy course during GA treatment (2002-2004)

Starting from March 2002, 3 months before the first MS relapse and initiation of GA treatment, the patient complained of recurrent stereotyped disorders consisting of different types of partial (simple and complex) seizures, lasting less than 3 minutes and sometimes overlapping: i) focal motor activity of the left part of the body, ii) loss of contact with surroundings followed by drowsiness, iii) gestural automatisms. Often, these episodes were followed by secondarily generalized tonic-clonic seizures (Figure 2A, Table 1). During 2002 increasing doses of carbamazepine were administered, whereas valproate daily dose was augmented to 1000 mg. Although the AED doses were enhanced, seizure frequency increased progressively even in the absence of MRI activity (Table 1 and Figure 2A). During 2004, despite treatment with valproate (1000 mg/day) and carbamazepine (1200 mg/day), the patient manifested 6 partial and 3 generalized seizures (Table 1 and Figure 2A).

#### c) Epilepsy worsening during GA treatment (2005-2008)

Notably, although the non-epileptic MS course was well controlled (one relapse in 2007; MRI stable in 2006), the epileptic condition worsened during the period 2005-2007 (Figure 2A, Table 1). Besides an increased number and duration (over 3 minutes) of partial and generalized seizures, the patients also manifested focal and generalized convulsive *status epilepticus *requiring intervention in intensive care department and treatment with intravenous lorazepam, diazepam and/or phenobarbital.

Starting from 2006, seizures were treated with other AEDs (including topiramate, levetiracetam, oxcarbazepine and gabapentin) at increasing daily dosage and often in association, as indicated on Table 1. Partial epileptic discharges at EEG were commonly observed (data not shown). Despite the frequent therapy changes in dose and type of AEDs, the pharmacologic control of seizures was clearly ineffective and the patient was diagnosed with refractory MS-related epilepsy in late 2006.

In 2008, the patient manifested at least 10 documented partial and 7 generalized seizures. In June 2008 he was brought to the intensive care department for generalized *status epilepticus*. A month later, an intercritical EEG showed partial epileptic discharges (Figure 2B). Although in October 2008 we documented a new MS relapse, we cannot exclude a contribution of the elevated AEDs daily dosage in the decline of cognitive status at the end of 2008 [6].

#### d) Effect of Natalizumab on epilepsy

Natalizumab therapy was initiated in February 2009 and till January 31, 2010 (12 months follow-up) the patient manifested only 4 short (less than 2 minutes) complex partial seizures (60% reduction compared to 2008). Importantly, during natalizumab treatment we observed no generalized seizures (100% reduction) and the absence of status epilepticus. Intercritical EEG performed in June 2009 was normal (Figure 2C). Oxcarbazepine was progressively discontinued, and the patient was treated with gabapentin 900 mg/day and levetiracetam, which was progressively reduced (1000 mg/day). Although there are no known interactions between natalizumab and AEDs, further studies are warranted to formally exclude this possibility. Overall, the results showed a dramatic improvement of seizure activity after 12 months of treatment with natalizumab.

## Discussion

Epilepsy affects between 0.5-1% of world population and, despite medical therapy, about one third of patients develop refractory epilepsy [16-18]. Among other factors, MS represents a risk factor for refractory epilepsy [19]. The pathophysiology of seizures in MS remains to be elucidated although cortical and subcortical lesions (as in our case, Figure 1C) may reasonably explain their increased frequency in MS [5]. Seizures can occur in MS patients as the presenting symptom or as a relapse, being either related or unrelated to other, non epileptic clinical relapses [5]. In our case, seizures occurred in both conditions as indicated on Table 1. Epileptic and non-epileptic symptoms were concomitant at disease onset (seizures followed ophthalmoparesis by 24 hours), and during 2004, and 2007. In contrast, seizures appeared to be clinically and temporally isolated in 2001, 2002, 2003, 2005, 2006 and 2008 and during the course of natalizumab therapy.

Experimental and clinical studies have shown that inflammation mechanisms are activated in epilepsy [7]. Proinflammatory cytokines such as IL-1β, TNF-α and IL-6 have been shown to be overexpressed in experimental models of seizures, prominently by glia [20], suggesting that glia activation may contribute to vascular inflammation in epilepsy. In addition, increased proinflammatory cytokines were found in the serum and CSF in patients with epilepsy, whereas the analysis of human brain specimens from drug-refractory epileptic patients showed strong activation of the IL-1b/IL-1R1 system in brain resident cells, such as in glia and neurons [21,22]. Functional interactions between cytokines and classical neurotransmitters such as glutamate and GABA have been also described, pointing out at novel glio-neuronal communications in epilepsy and suggesting that cytokine-mediated changes in neuronal excitability may promote seizures [20]. Innate immune mechanisms such as complement have been also shown to be potentially involved in epilepsy [22]. The role of innate immunity was further supported by a recent study performed in spontaneously epileptic mice and in human TLE showing a new proconvulsant pathway involving high-mobility group box-1 (HMGB1) release from neurons and glia and its interaction with Toll-like receptor 4 (TLR4) [23]. TLR4 and HMGB1 blockade by pharmacologic inhibitors or by genetic deficiency, potentially interrupted glia-neuron communication and reduced seizure generation in animal models of seizures in this study [23].

In addition to the inflammation mechanisms described above, recent studies performed in experimental animal models with relevance to human disease show a role for vascular inflammatory mechanisms and leukocyte-endothelial adhesion in the induction of BBB leakage and seizure generation [10,11]. BBB breakdown has been implicated both in the induction of seizures and in the progression to epilepsy with chronic seizure generation by exposure of neuronal cells to blood albumin and potassium ions [9,24-26]. Moreover, inhibition of BBB breakdown by blockade of leukocyte-endothelial interaction with an anti-VLA-4 antibody has preventive as well as therapeutic effects in a mouse model of epilepsy [10]. VLA-4 mediates adhesion of lymphocytes [27], monocytes [28] and under inflammatory conditions also of neutrophils [29], suggesting that leukocytes from innate and adaptive immunity may both contribute to seizure generation.

Acute seizure activity induces expression of adhesion molecules on brain endothelium [10,30]. Importantly, it has been recently shown that also recurrent seizures lead to chronic expression of VCAM-1, the ligand for VLA-4 integrin, potentially contributing to BBB permeability, neuroinflammation and brain damage potentially contributing to the evolution of chronic disease [10]. Vascular inflammation induced by each seizure (eventually also in the absence of concomitant infection and autoimmunity) may allow adhesion and transmigration of myeloid cells and activated lymphocytes, increasing local inflammation and potentially favoring the generation of new seizures. However, whether vascular inflammation, leukocyte trafficking mechanisms and BBB leakage are involved in all types of epilepsy need to be clarified in further studies.

In support to the results obtained from animal models of epilepsy, it has been shown that BBB disruption in patients with cerebral lymphoma induces focal motor seizures [9]. Vascular alterations and lymphocyte accumulation into the brain parenchyma were documented in a study performed on 87 young patients with refractory epilepsy [12]. In addition, perivascular and parenchymal T lymphocytes with a predominance of CD8 cytotoxic cells were found in grey and white matter in samples obtained from patients with tuberous sclerosis complex [31]. Cells of the microglia/macrophage cell system and scarce CD3 lymphocytes were also found to accumulate in brain samples obtained from patients with of TLE and hippocampal sclerosis [21]. In addition, recent results showed increased number of leukocytes in brain parenchyma of patients with epilepsy independently on the disease etiology [10]. In line with these previous data showing that leukocyte subpopulations may accumulate in the brain of patients with epilepsy, the results described in the present manuscript show successful treatment of epilepsy with natalizumab in a patient with MS. Notably, MS and epilepsy started concomitantly, but disease courses were relatively divergent during a 6-year period in which MS was under relative control with GA treatment whereas seizure frequency, duration and severity highly increased. Interestingly, generalized seizures preceded most of the new MS relapses leading us to speculate that, as seizure activity *per se *induces vascular inflammation [10], seizure activity may contribute to MS worsening. Thus, our results suggest that MS contribution to epilepsy induction, together with seizure activity potentially favoring MS relapses, may create a positive feedback loop between the two disease conditions.

The generalization of the remarkable effect of natalizumab on refractory epilepsy observed in the present clinical case to other more common types of epilepsy requires further studies. In contrast to the anti-adhesion therapy with natalizumab, a specific and sustained anti-adhesive activity exerted by GA and steroids in the present clinical case seems rather unlikely. In fact, GA treatment causes in vivo changes of the cytokine secretion pattern and effector function of GA-specific T cells but increases the migration rate of Th2 cells and do not affect the migration of Th1 cells [32,33], whereas steroids may reduce endothelial activation in a transient and non-selective manner during its short clinical use after MS relapses.

## Conclusions

Current pharmacological treatments for epilepsy do not address the inflammatory component of pathogenesis highlighted by recent studies and most AED drugs aim to depress aberrant neuronal excitation. However, noncompliant and refractory epilepsy cases demand investigation into alternative mechanisms and corresponding treatments, and interfering with the adhesion of immune cells to the cerebral vasculature may potentially open new avenues for epilepsy treatment [34].

Our results indicate treatment with natalizumab as highly effective in patients with MS and epilepsy. As no interactions with AED drugs were described for natalizumab and recent clinical data show that safety may be increased for PML associated with natalizumab therapy [35], our results suggest that anti-adhesion therapies such as natalizumab may complement traditional therapies, and might be useful in treating refractory epilepsy and epilepsy following MS or inflammatory inciting events such as trauma, stroke and infections.

Taking into account our current understanding of the pathogenesis of seizures and epilepsy and the emerging key role of inflammation mechanisms in epilepsy, our data suggest a more general application of natalizumab and anti-adhesion therapy in other types of epilepsy.

## Competing interests

Only public funds from the University of Sassari and the University of Verona were used for the present paper. No conflicts of interest exist with the companies whose products are mentioned and/or discussed in this article. GC is co-author in a patent owned by Stanford University USA and entitled "Anti-leukocyte recruitment therapy for the treatment of seizures and epilepsy" (U.S. Patent Application Serial No. 11/811,245).

## Authors' contributions

All authors fulfill the authorship criteria because of their substantial contributions to the conception, design, analysis and interpretation of the data. SS and GC wrote the manuscript, MRM made the clinical follow-up of the patient. All authors gave their final approval of the version to be published.

## Consent

Written informed consent was obtained from the patient for publication of this case report and any accompanying images. A copy of the written consent is available for review by the Editor-in-Chief of this journal.

## Pre-publication history

The pre-publication history for this paper can be accessed here:

http://www.biomedcentral.com/1471-2377/10/84/prepub

## References

[B1] Noseworthy JH Lucchinetti C Rodriguez M Weinshenker BG Multiple sclerosis N Engl J Med 2000 343 938 952 10.1056/NEJM200009283431307 11006371

[B2] McFarland HF Martin R Multiple sclerosis: a complicated picture of autoimmunity Nat Immunol 2007 8 913 919 10.1038/ni1507 17712344

[B3] Stone LA Smith ME Albert PS Bash CN Maloni H Frank JA McFarland HF Blood-brain barrier disruption on contrast-enhanced MRI in patients with mild relapsing-remitting multiple sclerosis: relationship to course, gender, and age Neurology 1995 45 1122 1126 7783875 10.1212/wnl.45.6.1122

[B4] Polman CH Reingold SC Edan G Filippi M Hartung HP Kappos L Lublin FD Metz LM McFarland HF O'Connor PW Sandberg-Wollheim M Thompson AJ Weinshenker BG Wolinsky JS Diagnostic criteria for multiple sclerosis: 2005 revisions to the "McDonald Criteria" Ann Neurol 2005 58 840 846 10.1002/ana.20703 16283615

[B5] Koch M Uyttenboogaart M Polman S De Keiser J Seizures in multiple sclerosis Epilepsia 2008 49 948 953 10.1111/j.1528-1167.2008.01565.x 18336559

[B6] Zaccara G Neurological comorbidity and epilepsy: implications for treatment Acta Neurol Scand 2009 120 1 15 10.1111/j.1600-0404.2008.01146.x 19527225

[B7] Vezzani A Granata T Brain inflammation in epilepsy: experimental and clinical evidence Epilepsia 2005 46 1724 1743 10.1111/j.1528-1167.2005.00298.x 16302852

[B8] Ransohoff RM Immunology: Barrier to electrical storms Nature 2009 457 155 156 10.1038/457155a 19129836

[B9] Marchi N Angelov L Masaryk T Fazio V Granata T Hernandez N Hallene K Diglaw T Franic L Najm I Janigro D Seizure-promoting effect of blood-brain barrier disruption Epilepsia 2007 48 732 742 10.1111/j.1528-1167.2007.00988.x 17319915 PMC4135474

[B10] Fabene PF Navarro Mora G Martinello M Rossi B Merigo F Ottoboni L Bach S Angiari S Benati D Chakir A Zanetti L Schio F Osculati A Marzola P Nicolato E Homeister JW Xia L Lowe JB McEver RP Osculati F Sbarbati A Butcher EC Constantin G A role for leukocyte-endothelial adhesion mechanisms in epilepsy Nat. Med 2008 14 1377 1383 10.1038/nm.1878 19029985 PMC2710311

[B11] Kim JV Kang SS Dustin ML McGavern DB Myelomonocytic cell recruitment causes fatal CNS vascular injury during acute viral meningitis Nature 2009 457 191 195 10.1038/nature07591 19011611 PMC2702264

[B12] Hildebrandt M Amann K Schröder R Pieper T Kolodziejczyk D Holthausen H Buchfelder M Stefan H Blümcke I White matter angiopathy is common in pediatric patients with intractable focal epilepsies Epilepsia 2008 49 804 815 10.1111/j.1528-1167.2007.01514.x 18266747

[B13] Steinman L Blocking adhesion molecules as therapy for multiple sclerosis: natalizumab Nature Rev Drug Discov 2005 4 510 519 10.1038/nrd1752 15931259

[B14] Steinman L A molecular trio in relapse and remission in multiple sclerosis Nat Rev Immunol 2009 9 440 447 10.1038/nri2548 19444308

[B15] Kurtzke JF Rating neurological impairment in multiple sclerosis: an expanded disability status scale (EDSS) Neurology 1983 33 1444 1452 6685237 10.1212/wnl.33.11.1444

[B16] Sander J The epidemiology of epilepsy revisited Curr Opin Neurol 2003 16 165 170 10.1097/00019052-200304000-00008 12644744

[B17] Forsgren L Beghi E Oun A Sillanpaa M The epidemiology of epilepsy in Europe- a systematic review Eur J Neurol 2005 12 245 253 10.1111/j.1468-1331.2004.00992.x 15804240

[B18] Beleza P Refractory epilepsy: a clinically oriented review Eur Neurol 2009 62 65 71 10.1159/000222775 19521080

[B19] Kelley BJ Rodriguez M Seizures in patients with multiple sclerosis: epidemiology, pathophysiology and management CNS Drugs 2009 23 805 815 10.2165/11310900-000000000-00000 19739692 PMC2748351

[B20] Vezzani A Balosso S Ravizza T The role of cytokines in the pathophysiology of epilepsy Brain Behav Immun 2008 22 797 803 10.1016/j.bbi.2008.03.009 18495419

[B21] Ravizza T Gagliardi B Noé F Boer K Aronica E Vezzani A Innate and adaptive immunity during epileptogenesis and spontaneous seizures: evidence from experimental models and human temporal lobe epilepsy Neurobiol Dis 2008 29 142 160 10.1016/j.nbd.2007.08.012 17931873

[B22] Aronica E Boer K van Vliet EA Redeker S Baayen JC Spliet WG van Rijen PC Troost D da Silva FH Wadman WJ Gorter JA Complement activation in experimental and human temporal lobe epilepsy Neurobiol Dis 2007 26 497 511 10.1016/j.nbd.2007.01.015 17412602

[B23] Maroso M Balosso S Ravizza T Liu J Aronica E Iyer AM Rossetti C Molteni M Casalgrandi M Manfredi AA Bianchi ME Vezzani A Toll-like receptor 4 and high-mobility group box-1 are involved in ictogenesis and can be targeted to reduce seizures Nat Med 2010 16 413 419 10.1038/nm.2127 20348922

[B24] Seiffert E Dreier JP Ivens S Bechmann I Tomkins O Heinemann U Friedman A Lasting blood-brain barrier disruption induces epileptic focus in the rat somatosensory cortex J Neurosci 2004 24 7829 7836 10.1523/JNEUROSCI.1751-04.2004 15356194 PMC6729929

[B25] Ivens S Kaufer D Flores LP Bechmann I Zumsteg D Tomkins O Seiffert E Heinemann U Friedman A TGF-beta receptor-mediated albumin uptake into astrocytes is involved in neocortical epileptogenesis Brain 2007 130 535 547 10.1093/brain/awl317 17121744

[B26] van Vliet EA da Costa Araújo S Redeker S van Schaik R Aronica E Gorter JA Blood-brain barrier leakage may lead to progression of temporal lobe epilepsy Brain 2007 130 521 534 10.1093/brain/awl318 17124188

[B27] Alon R Kassner PD Carr MW Finger EB Hemler ME Springer TA The integrin VLA-4 supports tethering and rolling in flow on VCAM-1 J Cell Biol 1995 128 1243 1253 10.1083/jcb.128.6.1243 7534768 PMC2120426

[B28] Ramos CL Huo Y Jung U Ghosh S Manka DR Sarembock IJ Ley K Direct demonstration of P-selectin- and VCAM-1-dependent mononuclear cell rolling in early atherosclerotic lesions of apolipoprotein E-deficient mice Circ Res 1999 84 1237 1244 10364560 10.1161/01.res.84.11.1237

[B29] Johnston B Kubes P The alpha4-integrin: an alternative pathway for neutrophil recruitment? Immunol Today 1999 20 545 550 10.1016/S0167-5699(99)01544-3 10562704

[B30] Librizzi L Regondi MC Pastori C Frigerio S Frassoni C de Curtis M Expression of adhesion factors induced by epileptiform activity in the endothelium of the isolated guinea pig brain in vitro Epilepsia 2007 48 743 751 10.1111/j.1528-1167.2007.01047.x 17386052

[B31] Boer K Jansen F Nellist M Redeker S van den Ouweland AM Spliet WG van Nieuwenhuizen O Troost D Crino PB Aronica E Inflammatory processes in cortical tubers and subependymal giant cell tumors of tuberous sclerosis complex Epilepsy Res 2008 78 7 21 10.1016/j.eplepsyres.2007.10.002 18023148

[B32] Schrempf W Ziemssen T Glatiramer acetate: mechanisms of action in multiple sclerosis Autoimmun Rev 2007 6 469 75 10.1016/j.autrev.2007.02.003 17643935

[B33] Prat A Biernacki K Antel JP Th1 and Th2 lymphocyte migration across the human BBB is specifically regulated by interferon beta and copolymer-1 J Autoimmun 2005 24 119 24 10.1016/j.jaut.2005.01.004 15829404

[B34] Kleen JK Holmes GL Brain inflammation initiates seizures Nat Med 2008 14 1377 83 10.1038/nm1208-1309 19057551

[B35] Wenning W Haghikia A Laubenberger J Treatment of progressive multifocal leukoencephalopathy associated with natalizumab N Engl J Med 2009 361 1075 1080 10.1056/NEJMoa0810257 19741228

